# Supplementary material for: Polyketide synthase gene clusters in the ericoid mycorrhizal fungus Oidiodendron maius: genome-wide prediction, expression profiling, and functional characterization of knockout mutants
Source: Front Plant Sci. 2026 Sep 16;17:1924430. doi: 10.3389/fpls.2026.1924430 (PMC13623903; doi:10.3389/fpls.2026.1924430)
Supplement: Supplementary file 1 [file SupplementaryFile1.zip › Supplementary Materials/Supplementary Folder 1.zip!/Oidma1_AssemblyScaffolds_Repeatmasked/knownclusterblast/cluster12/jgi.p_Oidma1_157867_mibig_hits.html]

| MIBiG Protein | Description | MIBiG Cluster | MiBiG Product | % ID | % Coverage | BLAST Score | E-value |
| --- | --- | --- | --- | --- | --- | --- | --- |
| EED17999.1 | benzoate\_4-monooxygenase\_cytochrome\_P450,\_putative | BGC0000154 | Stipitatic\_acid\_biosynthetic\_gene\_cluster | 58 | 73.5124760077 | 448 | 8.1e-126 |
| CDM36724.1 | Cytochrome\_P450 | BGC0001360 | Mycophenolic\_acid\_biosynthetic\_gene\_cluster | 51 | 94.6257197697 | 501 | 1.1e-141 |
| ADY00131.1 | putative\_P450\_monooxygenase | BGC0000104 | Mycophenolic\_acid\_biosynthetic\_gene\_cluster | 42 | 68.7140115163 | 265 | 9.7e-71 |
| EAQ84780.1 | hypothetical\_protein | BGC0001219 | Shanorellin\_biosynthetic\_gene\_cluster | 48 | 43.3781190019 | 215 | 1.2e-55 |
| ADI24958.2 | GsfF | BGC0000070 | Griseofulvin\_biosynthetic\_gene\_cluster | 29 | 97.1209213052 | 209 | 8.3e-54 |
| NP\_199072.1 | cytochrome\_P450,\_family\_705,\_subfamily\_A,\_polypeptide\_12 | BGC0000669 | Marneral\_biosynthetic\_gene\_cluster | 28 | 40.4990403071 | 77 | 6.4e-14 |
| AEE83585.1 | cytochrome\_P450,\_family\_705,\_subfamily\_A,\_polypeptide\_1 | BGC0001313 | Arabidiol\_/\_Baruol\_biosynthetic\_gene\_cluster. | 28 | 34.9328214971 | 76 | 8.4e-14 |
| AGC83578.1 | P450\_monooxygenase | BGC0000818 | Notoamide\_biosynthetic\_gene\_cluster | 26 | 37.4280230326 | 75 | 2.4e-13 |
| NP\_199610.1 | cytochrome\_P450\_705A5 | BGC0000670 | Thalianol\_biosynthetic\_gene\_cluster | 28 | 35.1247600768 | 74 | 3.2e-13 |
| BAV32149.1 | cytochrome\_P450\_monooxygenase | BGC0001373 | Sordarin\_biosynthetic\_gene\_cluster | 28 | 38.7715930902 | 72 | 2.1e-12 |
| AEE83587.1 | cytochrome\_P450,\_family\_705,\_subfamily\_A,\_polypeptide\_2 | BGC0001313 | Arabidiol\_/\_Baruol\_biosynthetic\_gene\_cluster. | 26 | 33.5892514395 | 62 | 1.2e-09 |
